# Analysis of Activity-Dependent Energy Metabolism in Mice Reveals Regulation of Mitochondrial Fission and Fusion mRNA by Voluntary Physical Exercise in Subcutaneous Fat from Male Marathon Mice (DUhTP)

**DOI:** 10.3390/cells9122697

**Published:** 2020-12-16

**Authors:** Julia Brenmoehl, Daniela Ohde, Christina Walz, Martina Langhammer, Julia Schultz, Andreas Hoeflich

**Affiliations:** 1 Institute for Genome Biology, Leibniz-Institute for Farm Animal Biology (FBN), Wilhelm-Stahl-Allee 2, 18196 Dummerstorf, Germany; brenmoehl@fbn-dummerstorf.de (J.B.); ohde@fbn-dummerstorf.de (D.O.); walz@fbn-dummerstorf.de (C.W.); 2 Lab Animal Facility, Leibniz-Institute for Farm Animal Biology (FBN), Wilhelm-Stahl-Allee 2, 18196 Dummerstorf, Germany; martina.langhammer@fbn-dummerstorf.de; 3 Institute of Medical Biochemistry and Molecular Biology, University of Rostock, Schillingallee 70, 18057 Rostock, Germany; julia.schultz@med.uni-rostock.de

**Keywords:** mitochondrial fission and fusion, voluntary activity, subcutaneous fat, DUhTP mice

## Abstract

Physical inactivity is considered as one of the main causes of obesity in modern civilizations, and it has been demonstrated that resistance training programs can be used to reduce fat mass. The effects of voluntary exercise on energy metabolism are less clear in adipose tissue. Therefore, the effects of three different voluntary exercise programs on the control of energy metabolism in subcutaneous fat were tested in two different mouse lines. In a cross-over study design, male mice were kept for three or six weeks in the presence or absence of running wheels. For the experiment, mice with increased running capacity (DUhTP) were used and compared to controls (DUC). Body and organ weight, feed intake, and voluntary running wheel activity were recorded. In subcutaneous fat, gene expression of browning markers and mitochondrial energy metabolism were analyzed. Exercise increased heart weight in control mice (p < 0.05) but significantly decreased subcutaneous, epididymal, perinephric, and brown fat mass in both genetic groups (p < 0.05). Gene expression analysis revealed higher expression of browning markers and individual complex subunits present in the electron transport chain in subcutaneous fat of DUhTP mice compared to controls (DUC; p < 0.01), independent of physical activity. While in control mice, voluntary exercise had no effect on markers of mitochondrial fission or fusion, in DUhTP mice, reduced mitochondrial DNA, transcription factor Nrf1, fission- (Dnm1), and fusion-relevant transcripts (Mfn1 and 2) were observed in response to voluntary physical activity (p < 0.05). Our findings indicate that the superior running abilities in DUhTP mice, on one hand, are connected to elevated expression of genetic markers for browning and oxidative phosphorylation in subcutaneous fat. In subcutaneous fat from DUhTP but not in unselected control mice, we further demonstrate reduced expression of genes for mitochondrial fission and fusion in response to voluntary physical activity.

## 1. Introduction

Physical exercise can have positive effects on health by reducing body or fat mass. On the cellular or molecular level in fat cells, even moderate or voluntary mild exercise may induce specific adaptations inducing the formation of beige fat, thermogenesis, or fat mobilization with the consecutive reduction of body fat [1,2,3]. Beige adipose tissue is characterized by increased mitochondrial biogenesis induced by peroxisome-proliferator-activated receptor-gamma-coactivator-1-alpha (PGC1-α) in muscle and fat, leading to an increase in mitochondrial mass, proteins, and capacity [4]. Elevated protein and mRNA levels of PGC1-α can be observed in various tissues of mice and rats in response to physical activity, particularly after endurance training [5,6,7]. In addition, Pgc1-α expression was induced by voluntary running wheel activity in subcutaneous fat [8,9] or in brown adipose tissue [1]. In subcutaneous fat, voluntary physical activity resulted in elevated mRNA expression of genes involved in PGC1-α-related pathways, including transcription factors nuclear respiratory factor 1 (Nrf1) and mitochondrial transcription factor A (Tfam) [9], inducing the transcription of mitochondrial genes. That, in turn, led to an induction of browning/beiging, and mitochondrial biogenesis [3,9] or an improved mitochondrial network [8].

It is known that mitochondria undergo dynamics that include biogenesis, fission, fusion, depolarization, and autophagy [10,11,12] to ensure the exchange of organelle content and meet the energy demand of the tissue [13]. Mitochondrial fusion is mainly induced by mitofusin 1 (MFN1) and mitofusin 2 (MFN2) for outer mitochondrial membrane fusion and the optical protein atrophy 1 (OPA1) protein for inner mitochondrial membrane fusion. On the contrary, mitochondrial fission 1 protein (FIS1) and dynamin-related protein 1 (DNM1) play a decisive role in mitochondrial fission [14,15]. Since muscle PGC1-α regulates the expression of Mfn1 and 2 [16] and mitophagy in response to acute stress [17], a direct relationship was assumed between the transcriptional regulation of mitochondrial biogenesis and mitochondrial dynamics in the muscle [10]. In response to acute stress, mitochondrial fission appears to be favored in skeletal muscle [18], as reduced muscular mitofusin1 protein expression and fission-related FIS1 protein increase were detected in rats up to 48 h after the last exercise bout [19,20], and DRP1 activation was induced in mice [21]. Whereas increased mitochondrial fusion appears to predominate as a result of prolonged training in muscle [22]. For example, C57BL/6J mice active in the running wheel for 30 days exhibited similar levels of proteins involved in mitochondrial fusion and fission but had elevated levels of phosphorylated dynamin-related protein 1 (DRP1; gene *Dnm1*), which reduced its translocation and thus mitochondrial fission [23]. In subcutaneous fat, mitochondrial dynamics especially in response to voluntary activity, have been less well studied, to date.

The mouse model DUhTP (Dummerstorf mice selected for high treadmill performance) is characterized by beige subcutaneous fat already in a sedentary condition due to long-term selection for high treadmill performance [8,24,25]. Since the selection was based on a single submaximal run with males after mating, a selection line was generated, exhibiting genetically high running ability without any previous training. Interestingly, the extraordinary endurance performance of the DUhTP animals did not correlate with elevated voluntary running wheel activity, which was not significantly different from the unselected controls (DUC mice) [8,24,26]. DUhTP mice have selection-related adaptations that indicate the efficient use of lipids for superior endurance performance. The increased fat storage under sedentary conditions, associated with increased hepatic lipid production [24], is efficiently reduced by voluntary physical activity, which could be attributed to both reduced deposition of additional fat (subcutaneous, epididymal, perinephric, and brown fat) and mobilization of existing fat [8].

Since we had observed strong effects of voluntary physical activity on lipid metabolism in subcutaneous fat from DUhTP mice [8,27], we wanted to test the effects of voluntary exercise on the control of energy metabolism in this tissue on the molecular level. Therefore, we tested the effects of three different exercise programs in a cross-over study design on energy metabolism in subcutaneous fat from marathon mice (DUhTP) and unselected controls (DUC). In particular, we analyzed the expression of browning markers, genes from the oxidative phosphorylation pathway, and the expression of genes regulating mitochondrial fission and fusion in response to voluntary exercise protocols in DUhTP and DUC mice. In the present manuscript, we discuss the effects of genetic selection and the effects of exercise on the control of mitochondrial energy metabolism in subcutaneous fat.

## 2. Materials and Methods

### 2.1. Animals and Cross-Over Study Design

**Figure 1:**
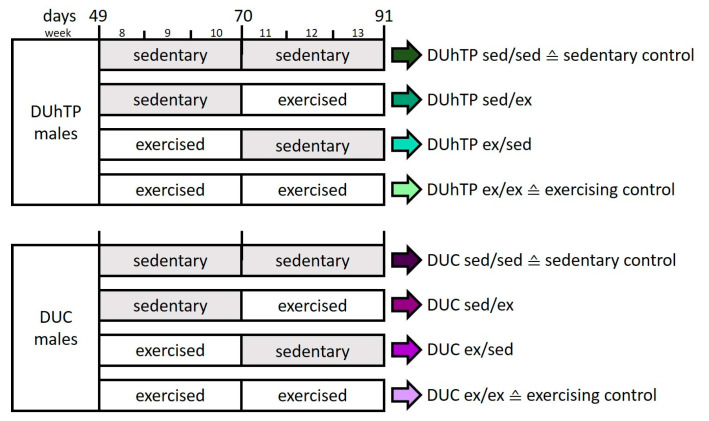
Voluntary exercise program for each experimental group. Male mice of the DUhTP and DUC lines were kept in individual cages without (sed) or with (ex) running wheel from 8 to 13 weeks of age (n = 8).

All in vivo experiments were conducted according to national and international guidelines and approved by our internal institutional review board. This study used mice selected from a broad genetic base population for high treadmill performance (DUhTP) over 140 generations [24,25] and unselected controls (DUC). Both mouse lines represent non-inbred lines, maintained by avoiding inbreeding. The animals were kept in H-Temp Polysulfon cages with a floor area of 370 cm^2^ (Eurostandard Type II, Tecniplast, Hohenpeißenberg, Germany) under controlled, specified pathogen-free (SPF) conditions. The mice were given fresh tap water and were fed autoclaved Ssniff^®^ M-Z feed (Ssniff-Spezialdiäten GmbH, Soest, Germany) ad libitum. Weekly feed intake and body weight development were monitored for 42 days in 7- to 13-week-old mice as described [24]. For the study, male full-sib animals kept in single cages from day 21 were divided into four groups (n = 8) at the age of 48 days and assigned to an individual voluntary exercise program (Figure 1). The animals of the sedentary control group were kept in individual cages for six weeks without any treatment (sed/sed). Whereas, the exercising control group had six weeks of access to a running wheel (Conventional Activity Wheel, diameter = 33.4 cm, equipped with a counter that recognized every quarter rotation; Tecniplast) in their cages (ex/ex). In a cross-over study design, the wheels were removed from one experimental group (ex/sed) after three weeks and placed in the cages of another experimental group that spent three weeks sedentary (sed/ex). Accordingly, groups sed/ex and ex/sed were sedentary in the first or in the second half of the six-week study period. The animals could use the wheel at will, and voluntary physical activity was determined based on running wheel activity (quarter rounds per day). The individual activity was monitored, whereby one complete rotation of the wheel (diameter = 33.4 cm) corresponded to a running distance of 1 m.

At the age of 13 weeks, mice were sacrificed, and serum samples were taken. Tissues were weighed, shock frozen in liquid nitrogen, and stored at −70 °C for subsequent analysis.

### 2.2. Analysis of Triglycerides in Serum Samples

Total triglyceride concentration was determined in serum samples using commercial kits (triglycerides: No. LT-TR 0015; Labor & Technik Eberhard Lehmann, Berlin, Germany) [24]. Serum was used undiluted and analyzed for triglyceride content according to the manufacturer’s recommendations.

### 2.3. mRNA Expression in Subcutaneous Fat

The complete inguinal subcutaneous fat was ground in liquid nitrogen. Total RNA was isolated using Trizol as described before [28]. cDNA synthesis followed the manufacturer’s protocol of the used commercial kit (GoScript™ Reverse Transcription System, Promega, Walldorf, Germany). Transcript abundance was measured in 96well format on LightCycler 480^®^ (Roche, Mannheim, Germany) using GoTaq^®^ qPCR Master Mix (Promega) with BRYT Green^®^ Dye according to manufacturers’ instructions. Primers used are listed in Table 1. A serial dilution of the cDNA pool was used for relative quantification. Recalculation and normalization were done using software DAG [29].

### 2.4. Mitochondrial Morphology in Adipose Tissue

Mitochondrial staining was performed as previously described [8,30]. Frozen tissue sections (5 µm) were stained with MitoTracker^®^ Deep Red (Molecular Probes^®^ Invitrogen, Darmstadt, Germany) for 30 min at 37 °C before fixation in ice-cold acetone (−20 °C) for 10 min and DAPI counterstaining (Vector Laboratories, Burlingame, CA, USA). Mitochondrial morphology was demonstrated using a confocal laser-scanning microscope Fluoview FV10i (Olympus, Hamburg, Germany).

### 2.5. Statistical Analysis

Data analysis and graphical representations were performed using GraphPad Prism 8.2.1 (GraphPad Software, San Diego, CA, USA). The data could be considered as approximately normally distributed; outliers identified by the statistical analysis package from GraphPad Prism 8.2.1 were removed from the data set. All data concerning running activity, feed intake, phenotypic data, and serum triglycerides were analyzed by one-way ANOVA, multiple comparisons. Body weights between the groups within a line and bodyweight development within the group as well as mRNA expression data were compared by two-way ANOVA, multiple comparisons. The effect of selection or physical exercise on mRNA expression was evaluated for statistical significance by unpaired t-test or one-way ANOVA, respectively. All results were displayed as means with their standard errors of the mean (SEM).

Pearson correlation coefficients were calculated to determine the relations between the expression of browning markers and different mitochondrial genes. The effects and differences were considered significant if p < 0.05.

## 3. Results

### 3.1. Running Wheel Performance and Food Consumption in Response to Differential Exercise Programs

**Figure 2:**
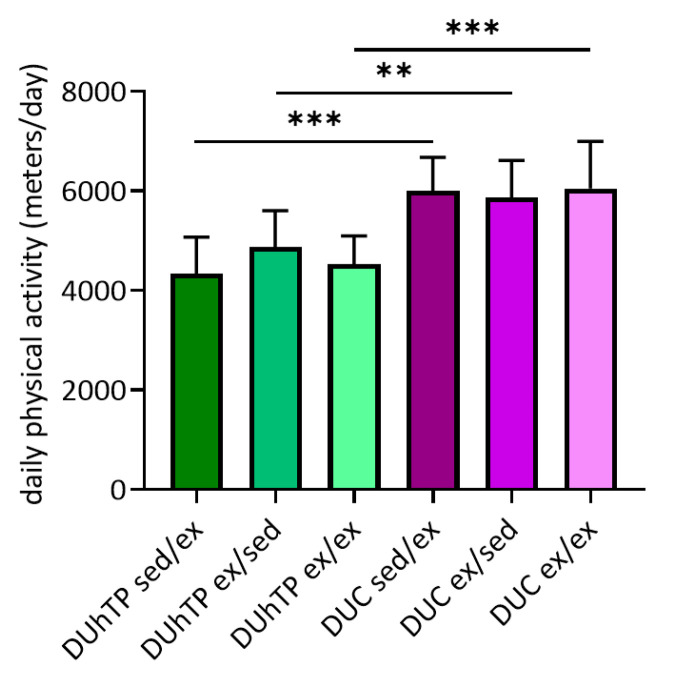
Voluntary physical activity of DUhTP and DUC males. The mice were kept from week 8 to 13, either in cages with or without included running wheels. Shown is the daily voluntary activity in meters for the respective running groups with the standard error of the mean ± SEM, n = 8. Significant differences were calculated by GraphPad, one-way ANOVA: ** p < 0.005, *** p < 0.0005.

The voluntary activity of each animal was recorded daily, and the average physical activity of the active groups was determined. The animals’ age and voluntary physical activity duration did not influence weekly or 3-weeks running performance (data not shown). However, line-specific differences were visible. While the groups DUhTP sed/ex, ex/sed, and ex/ex covered 4340 ± 737, 4877 ± 729, and 4530 ± 565 m per day, respectively, DUC sed/ex, ex/sed, and ex/ex mice ran 6002 ± 673, 5869 ± 745, 6051 ± 950 m per day (Figure 2). Accordingly, the DUC mice were characterized by a significantly higher voluntary running activity than the DUhTP mice (p < 0.005).

**Figure 3:**
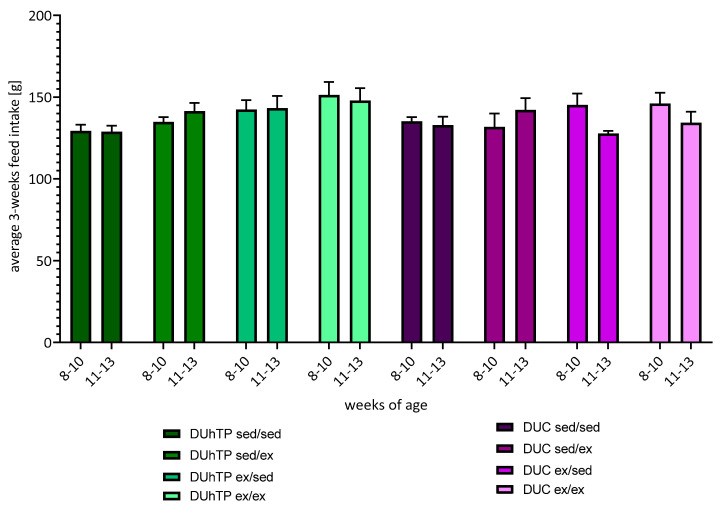
Feed intake of DUhTP and DUC mice from week 8 to 10 and 11 to 13. The food consumption was calculated by subtracting the remaining food from the weighed diet and displayed as average 3-weeks feed intake in grams ± SEM.

After six weeks of voluntary activity, feed intake increased by 11% (not significant) in DUhTP animals and by 4.6% (not significant) in DUC animals, compared to their respective sedentary control group (DUhTP ex/ex: 299.3 ± 11.2 g, DUhTP sed/sed: 264.8 ± 6.5 g; DUC ex/ex: 280.5 ± 11.2 g, DUC sed/sed: 268.2 ± 6.3 g). Also a 3-week comparison revealed no significant effect of physical activity on feed intake neither in DUhTP nor in DUC mice (Figure 3).

### 3.2. Effects of Three Different Exercise Programs on Body Weight, Serum Triglyceride Levels, and Fat Deposition

**Figure 4:**
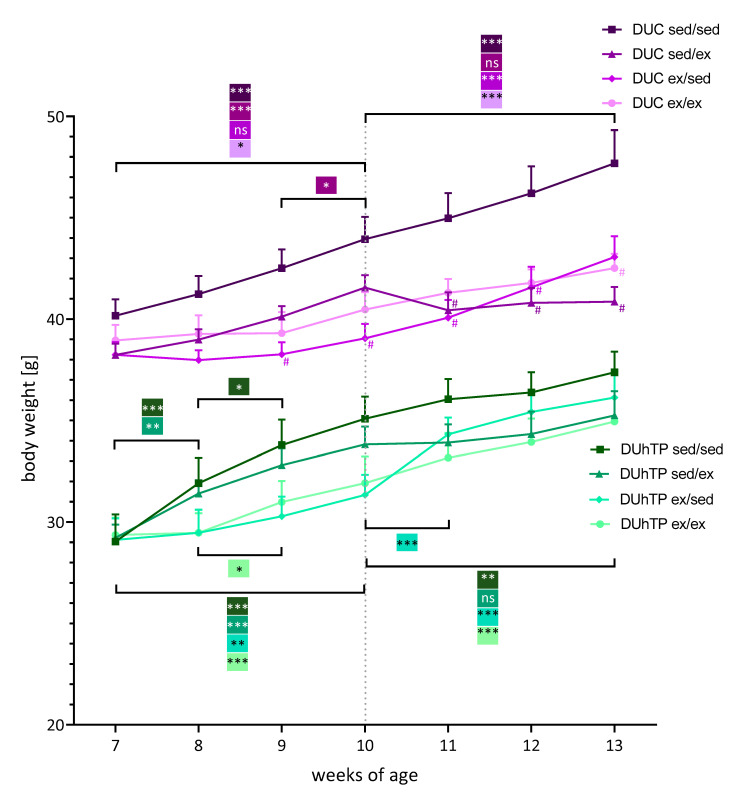
Effects of different exercise programs on body mass in DUhTP and DUC mice. Both lines’ animals were randomly attributed to four groups and individually kept with (ex) or without running wheel (sed) from week 8 to 10 or 11 to 13. The animals were weighed weekly, and the mean body mass ± SEM from eight animals per group are provided. Statistical analysis was performed by two-way ANOVA. Significance between two age points within one exercise group is depicted by a bracket, * p < 0.05, ** p < 0.005, *** p < 0.0005, ns, not significant; # p < 0.05 relative to respective sed/sed group at the same age.

The experimental groups of both genetic groups did not start with identical body weights in the beginnings of the study at day 49 (not significant; Figure 4). Body weights in DUC mice were approximately 33% higher than those of the DUhTP animals (DUhTP: 29.2 ± 2.7 g, DUC: 38.9 ± 2.1 g, p < 0.0001).

Within the first three weeks of the study, the body weights of sedentary DUhTP and DUC animals (sed/sed and sed/ex) increased to a greater extent than those of the animals, which had the opportunity to be physically active in a running wheel (ex/sed and ex/ex). A steady increase in body weight of about 3% per week was observed in the DUC sedentary controls. In contrast, the DUhTP sedentary controls increased significantly in weight, especially in the first two weeks (sed/sed: week 7–8: +9.9%, p < 0.0001; week 8–9: +5.9%, p < 0.05). After that, weight increased less from week to week but differed significantly between the beginning and the end of the 3-week interval (p < 0.005).

**Figure 5:**
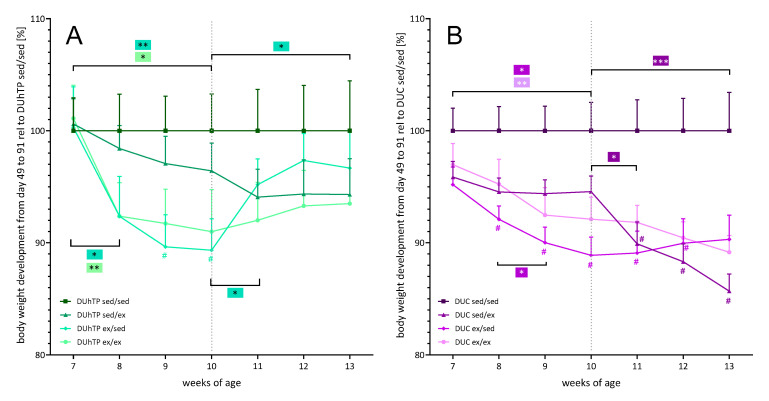
The relative weekly body weight developments of (**A**) DUhTP and (**B**) DUC animals with (ex) or without running wheels (sed) in the cage from week 8 to 10 or 11 to 13, by relating the body masses of the inactive control animals 100% and the determined body masses of the other groups. The body weights from eight animals per group ± SEM are provided. Statistical analysis was performed by two-way ANOVA. Significant differences between two age points within one exercise groups are depicted by brackets and color codes; * p < 0.05, ** p < 0.005, *** p < 0.0005; # p < 0.05 relative to the respective sed/sed group at the same age.

Interestingly, the body weight of the sedentary control (sed/sed) or exercising control (ex/ex) DUhTP mice increased steadily in the same manner with a weight difference of about 2–3 g, which were not significant due to individual variations within the exercise groups (Figure 5A). Instead, in the DUC exercising control group, mice gained weight more slowly than sedentary controls, resulting in significantly different body weights at week 13. Thus, permanent exercise slowed down body weight gain in DUC mice (Figure 4).

However, DUhTP ex/sed animals were characterized by a substantial increase in body weight (week 10–13: +15.3%, p < 0.0001), especially during the first week of inactivity (Figure 4; week 11: +3 g, p < 0.0001). Initially sedentary and then active DUhTP mice (sed/ex) showed a reduced body weight development from week 10 to 11 compared to the sedentary control DUhTP (sed/sed;) and then a weight development parallel to the sedentary control (Figure 5A).

DUC ex/sed mice also gained weight (+10.9%, p < 0.0005) due to inactivity in the second activity interval, although to a lower extent than the other line’s ex/sed animals. Thus, at week 13, no significant difference between the body weight of the DUC ex/sed and that of the DUC sedentary controls was found. Instead, it was observed that mice voluntarily active in the second interval (sed/ex) showed no increase in body weight (Figure 4, week 10–13: −0.7 g, n.s.). In relation to DUC sedentary controls, they exhibited a significantly diminished body weight development similar to the exercising control mice (Figure 5B).

**Figure 6:**
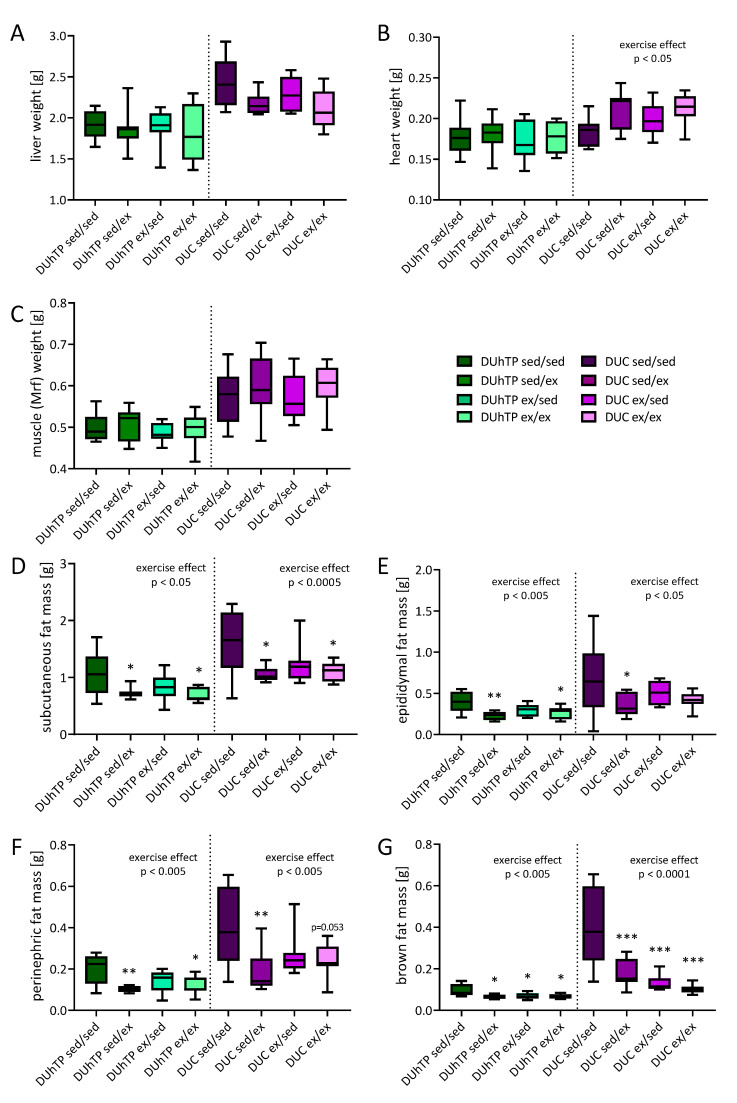
Absolute organ and tissue weights at week 13 of age in DUhTP and DUC mice in response to different activity programs. Both lines’ animals were attributed to four groups and individually kept from week 8 to 10 or 11 to 13, either with or without a running wheel. The absolut weights from (**A**) liver, (**B**) heart, and (**C**) muscle, as well as (**D**) subcutaneous, (**E**) epididymal, (**F**) perinephric, and (**G**) brown fat are presented as means ± SEM from eight animals per group. Statistical analysis was performed by two-way ANOVA. Significant effects of activity compared to the respective sedentary control as marked; * p < 0.05, ** p < 0.005, *** p < 0.0005. The overall effects of exercise are also stated.

At the age of 13 weeks, the animals were phenotypically examined in more detail. The liver, heart, and muscle mass (M. quadriceps femoris) were similar in all experimental groups of the respective mouse line (Figure 6A–C). However, it was shown that exercise, in general, had a positive effect on heart weight in DUC mice compared to sedentary control littermates (Figure 6B).

**Figure 7:**
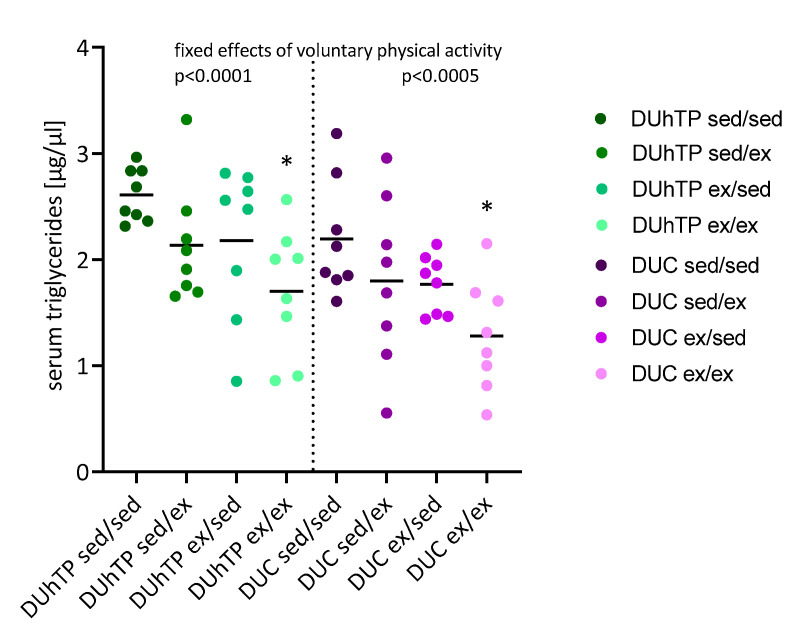
Serum triglyceride levels in 13-week-old exercised and sedentary DUhTP and DUC mice (n = 8). The mice were individually kept from week 8 to 10 or 11 to 13, either with or without a running wheel. Values are means of three technical replicates. Significant effects of six weeks of voluntary physical activity (ex/ex) compared to six weeks in sedentary conditions (sed/sed): * p < 0.05 (one-way ANOVA). The fixed effect of physical activity in general, as stated: p < 0.0005.

Robust effects of voluntary activity were observed on the fat depot weights (Figure 6D–G). In both genetic groups, voluntary activity, as a rule, decreased the weights of all fat depots examined (p < 0.05). In subcutaneous fat from DUhTP mice, six weeks of voluntary exercise led to reductions of 35% (ex/ex, p < 0.05), and three weeks of exercise in the second activity period resulted in a reduction of fat mass by 32% (sed/ex, p < 0.05) compared to the sedentary control group (sed/sed). Similar effects of physical activity were found in DUC mice with reductions of 30.9% (ex/ex, p < 0.05) and 33.6% (sed/ex, p < 0.05; Figure 6D). Also, significant effects were observed in epididymal, perinephric, and brown fat depots (Figure 6E–G), where voluntary physical activity decreased fat mass by up to 75%. In response to physical activity, serum concentrations of triglycerides were also decreased in both lines, however, to a significant extent only after six weeks of voluntary exercise (p < 0.05, Figure 7). In general, exercise significantly affected serum triglyceride levels in both lines (p < 0.0005).

### 3.3. Effects of Three Different Exercise Programs on the Expression of Browning Markers and Mitochondrial Genes

Before we started an in-depth molecular analysis of genetic markers for mitochondrial functions in subcutaneous adipose tissue in our experimental system, we conducted a preceding cross-sectional pilot study via a screening approach. We asked whether different conditions of the mitochondrial network can generally be assumed, or whether the mitochondrial network is identical in all experimental groups, which in the latter case would less strongly support further studies of energy metabolism in our study. Therefore, we chose one cryogenic section per experimental group and assessed the morphology of the mitochondrial networks, provided as supplementary material (Figure S1). These initial mitochondrial stainings revealed different conditions ranging from networks defined by a well-branched network of mitochondria in different groups to the presence of a mitochondrial network accompanied by massive clustering, fragmentation, and elongation of the mitochondria in one group. Since this pilot study, in fact, suggested the presence of different conditions of the mitochondrial network in different experimental groups, we next performed a detailed study of mitochondrial energy metabolism on the level of gene expression.

**Figure 8:**
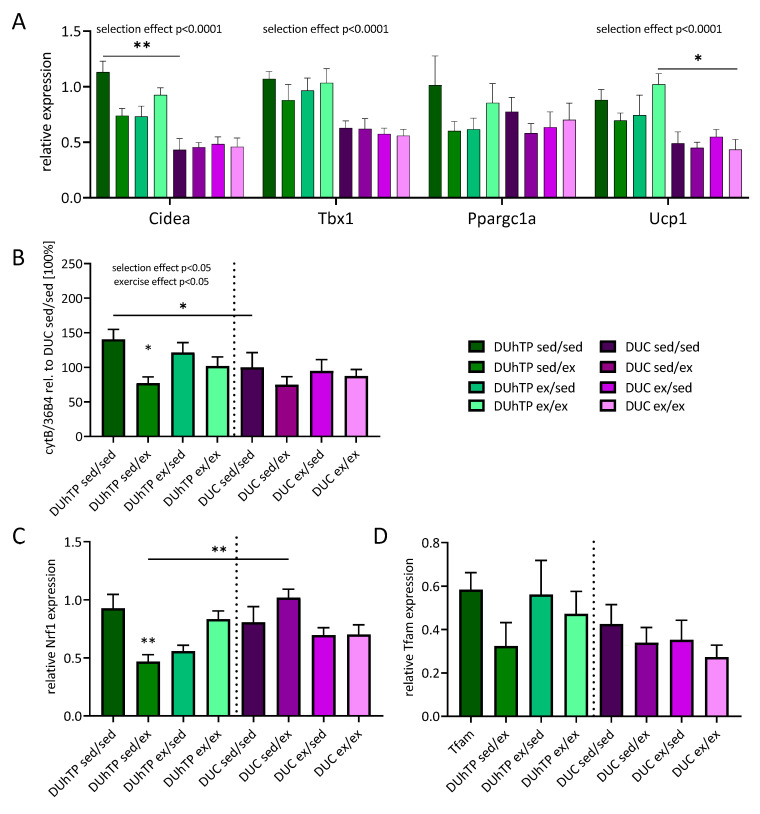
Analysis of mRNA expression in subcutaneous fat determined by quantitative real-time PCR in 13-weeks-old DUhTP and DUC mice after absolving different activity programs. The expression is normalized to housekeeping gene expression. Graphs show the relative expression of (**A**) browning markers, (**B**) ratio of mitochondrial to genomic DNA, and transcription factors (**C**) Nrf1 and (**D**) Tfam. Statistical analysis was performed by two-way ANOVA. Graphs show means (n = 8) with SEM. Significant effect of exercise compared to the respective sedentary line control as indicated by * p < 0.05, ** p < 0.005. Significant differences between the same exercise groups of both mouse lines are indicated by a line drawn over the bars and * p < 0.05 or ** p < 0.005. Overall effects of selection or exercise are also provided with the graphs.

Therefore, we first investigated the expression of browning markers in the context of the different exercise programs (Figure 8A). As a main effect of the genetic group and independent of physical activity, subcutaneous adipose tissue of DUhTP mice was characterized by elevated mRNA levels for Cidea, Tbx1, and Ucp1 (p < 0.0001) compared to unselected DUC mice. By comparing distinct treatment groups, sedentary control DUhTP mice had higher levels of Cidea mRNA transcripts in fat depots compared to DUC (p < 0.005). Besides, in DUhTP mice, six weeks of voluntary activity increased mRNA levels of Ucp1 in subcutaneous fat depots (p < 0.05) compared to the DUC mice from the identical treatment group. The three exercise interventions had no significant impact on Cidea, Tbx1, Ppargc1a, and Ucp1 mRNA expression. However, both lines showed a strong correlation between Cidea and Ucp1 expression, with no statistical significance for long-term active DUhTP (ex/ex) and short-term active DUC mice (sed/ex) (Table 2).

Since browning of adipose tissue is associated with mitochondrial biogenesis, we also studied mtDNA (ratio cytB/36B4) and the transcription factors Nrf1 and Tfam expression. Mitochondrial DNA expression was increased in the subcutaneous fat samples of sedentary control DUhTP mice compared to the DUC line (Figure 8B; p < 0.05). Interestingly, in DUhTP sed/ex mice, mtDNA expression was decreased (p < 0.05). Expression of the transcription factors Nrf1 and Tfam was also reduced in response to voluntary physical activity from week 10 to 13 (Figure 8C,D), although only the Nrf1 expression was significantly lower (Figure 8C; p < 0.005). DUC sed/ex mice expressed more subcutaneous fat Nrf1 transcripts than DUhTP mice (p < 0.005). Nevertheless, only for Tfam and Ppargc1a, a positive correlation could be shown in all exercised DUhTP animals and DUC sed/ex (Table 2).

**Figure 9:**
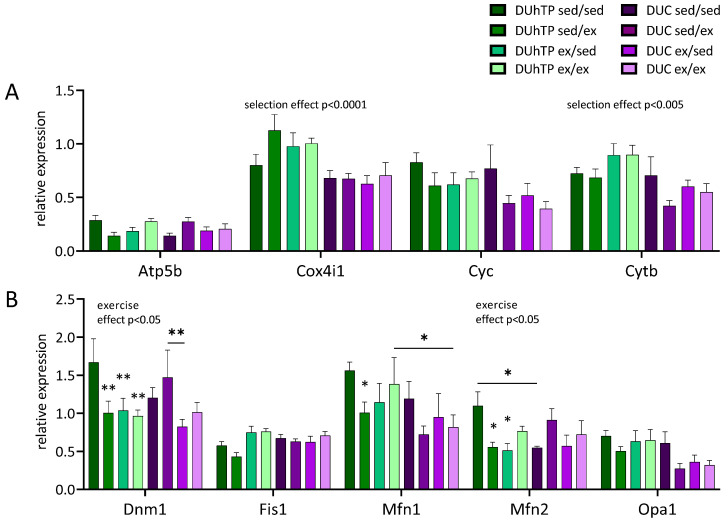
Analysis of mRNA expression in subcutaneous fat of 13-week-old DUhTP and DUC mice in response to different activity programs, determined by quantitative real-time PCR. The graphs show the relative expression (relative to the expression of the measured household genes) of genes related to (**A**) oxidative phosphorylation and (**B**) mitochondrial dynamics (fusion and cleavage). Statistical analysis was performed by two-way ANOVA. The graphs show the mean values (n = 8) with SEM. Significant effects of voluntary activity compared to the respective sedentary control, indicated by * p < 0.05, ** p < 0.005. Significant differences between two groups are indicated by a line over the bars and * p < 0.05 or ** p < 0.005. The main effects of selection and exercise in DUhTP are also shown in the graphs.

Surprisingly, gene expression of mitochondrial complex subunits showed no exercise-related changes (Figure 9A). However, an effect of selection was found for the complex subunits Cox4i1 (p < 0.0001) and Cytb (p < 0.05) as well as a positive correlation between Ppargc1a and Cytb and Cyc (Table 2).

Under the conditions of this experiment, an effect of selection could not be identified for any of the five genetic markers (Dnm1, Fis1, Mfn1, Mfn2, or Opa1) controlling mitochondrial fission or fusion in subcutaneous adipose tissue (Figure 9B). Instead, a main effect of voluntary exercise could be observed in DUhTP mice for Dnm1 and Mfn2 (p < 0.05). Significant effects of the exercise were also demonstrated if distinct experimental groups were compared with sedentary DUhTP mice (p < 0.05).

Notably, mRNA expression of three different fission or fusion markers was reduced by voluntary physical activity in subcutaneous fat samples of DUhTP mice (p < 0.05). An inhibitory effect of physical activity was not observed in control mice.

Besides, correlation studies revealed a strong association between Ppargc1a and Opa1 and Mfn1 in exercised DUhTP mice and only sporadically in DUC mice (Table 2). The strong relationship between Mfn1 and Opa1 could be shown for both lines.

## 4. Discussion

Beige fat thermogenesis plays an essential role in the regulation of body weight. The conversion of white to beige adipose tissue (called browning) is typically induced by cold exposure [31] or training [2]. Due to long-term selection for high submaximal treadmill running over 140 generations, DUhTP mice have beige subcutaneous fat even in sedentary conditions and without external induction [24].

In previous studies, we demonstrated that three weeks of voluntary running wheel exercise significantly reduced fat mass and increased browning markers and the mitochondrial network in subcutaneous fat, particularly in DUhTP mice [8,27]. Considering the prominent effects of voluntary physical activity in the previous study [8], we now investigated three different voluntary exercise programs on gene expression related to energy metabolism in DUhTP mice and DUC controls.

Unexpectedly, the physical activity of DUhTP mice was significantly lower compared to unselected controls. This result was different from our previous study in animals of earlier generations under conditions of a semi-barrier animal facility [8,24], where no significant difference of voluntary activity was described in the DUhTP and DUC mice. Meanwhile, the mice were brought to an SPF facility, and several genotype/environment interactions have been identified [26]. In particular, the new environment had a significant effect on growth and reproduction traits. At the time of the present study, voluntary physical activity in our model also seemed to be affected by the new environmental effects. We can therefore assume that voluntary physical activity is a dynamic trait in our model, which is currently being investigated in a separate study. Overall, the observed dynamic traits are related to the mouse model’s complex genetic background, initially composed of four different inbred and four different outbred mouse strains [32]. Moreover, as an effort to maintain genetic diversity in our mouse lines, inbreeding was avoided during the selection experiment. Within each line, voluntary physical activity was similar at all time points tested. This observation is different from a study by Davidson and colleagues [33]. They demonstrated increased running activity over time, although the running activity in DUhTP and DUC mice were on higher levels compared to the referenced study.

Voluntary physical activity blocked the age-related weight gain in both genetic groups in the first period of the 3-week segments compared to both sedentary control groups. However, the negative effects of exercise on body weight gain were no longer present after three weeks of physical inactivity, especially in DUhTP mice. When the sedentary mice of both genetic groups were allowed to use a running wheel in the second half of the study (sed/ex), weight gain between weeks 10 and 11 decreased in both genetic groups as well. While this effect persisted in the DUC animals until the end of the experiment, similar weight gain was observed in the DUhTP sed/ex animals from week 11 onwards as in the sedentary control siblings. The body weight differences of the sedentary controls compared to partially and long-term exercised animals were more pronounced in the DUC animals and reached statistical significance for animals that ran until the end of the experiment (sed/ex, ex/ex). Physical activity affected DUC animals much more strongly in the second half of the experiment resulting in the lowest bodyweight of this line. On the other hand, the DUhTP animals increased in weight due to forced inactivity during the second part of the experiment and hardly differed from sedentary control animals.

Comparable results were described in rats that stopped training four weeks earlier than their littermates, which trained for a total of 12 weeks [34]. After the resting period, these animals were also characterized by an increase in body weight, and their final body weight corresponded to the body mass of sedentary subjects. While food consumption was not investigated in this study [34], Tokuyama and colleagues demonstrated that physical activity correlates with increased feed intake compared to sedentary controls [35]. This finding was not confirmed using our experimental design.

However, it was clearly shown that under our experimental conditions, substantial reductions in body fat were present in response to voluntary physical activity, as described in a previous, slightly different experimental design with only a 3-week-exercise interval [8]. In the current study, we identified significant reductions in all examined fat depots in DUhTP and unselected controls, which voluntarily ran until the experiment’s end. Voluntary exercise, which started later, showed a more significant effect on fat depots in mice of both lines. The animals of this group were characterized by lower epididymal and perinephric fat depots than exercising control animals. Lean aged mice that underwent a 10-weeks voluntary exercise intervention also showed a better decrease in epididymal fat mass and average adipocyte area than half aged animals [36]. Lehnig et al. also showed significant reductions in five (interscapular and perirenal brown as well as interscapular, perigonadal, and inguinal white adipose tissue) of 14 investigated fat depots in older animals by voluntary 3-week running wheel activity [9]. With the chosen study design of 10-week-old animals and 3-week voluntary wheel activity, these animals corresponded to the experimental group sed/ex from our cross-over study, as they were also physically active between 10 and 13 weeks of age.

No significant changes in subcutaneous, perinephric, and epididymal fat depot mass were detected in the animals of both lines that stopped exercising in the second part of our experiment. Sertie et al. made similar observations with rats undergoing 12 weeks of treadmill training [34]. Early discontinuation of training after eight weeks followed by 4-weeks of sedentary caging resulted in subcutaneous and retroperitoneal fat mass weights similar to those of entirely untrained animals, while those of permanently trained siblings were reduced. Another trial with rats applied the so-called wheel-lock model, which provides a one-week blocking of the running wheels after three weeks of voluntary exercise. During the last week, a dramatic increase in fat mass, especially visceral and perirenal fat, was observed in rats with wheel-lock, in contrast to permanently active [37] or permanently active and sedentary rats [38]. Noteworthy, the authors of this study were able to stop this increase in body fat by administering AICAR, a pharmacological agent that activates the AMP kinase and, therefore, energy metabolism [38].

Brown fat depots were reduced in both lines under the influence of voluntary physical activity. The reduction of this thermogenic tissue and its thermogenic capacity was also observed in other exercise trials in rodent models [8,9,39]. These reductions are assumed to be an adaptation to regular, chronic physical activity to counteract the training-mediated increase in muscular heat production [39]. This seems to be a longer-lasting adaptation since a cessation of voluntary exercise in our animals did not regain brown adipose tissue mass. According to Aldiss and colleagues, the exercise-induced reduction and browning of subcutaneous fat could be an adaptive mechanism that increases the thermal potential of the reduced brown but also subcutaneous fat mass and thus may compensate for the elevated sensation of cold [40].

Serum triglyceride levels were reduced by voluntary physical activity in both lines, and significant differences were observed after six weeks of physical activity. When quantified directly after three weeks of voluntary physical activity at the age of 10 weeks, a significant reduction of serum triglyceride concentration was identified in the DUhTP mouse model [8]. Thus, three weeks of physical inactivity could have normalized serum triglyceride levels in DUhTP mice at the age of 13 weeks in the present study. Since three weeks of voluntary physical activity between 10 and 13 weeks of age had no effect on serum triglycerides, we may argue that the effects of physical activity on serum triglycerides may depend on age. In fact, the triglyceride levels in serum from sedentary animals in both lines at the age of 13 weeks were twice as high if compared to 10 weeks of age and similar (DUC) or almost similar (DUhTP) to mice at the age of 7 weeks [24]. Since lipid metabolism, without a doubt, is required for aerobic energy metabolism during exercise in mice [41], the age-dependency of serum triglycerides in DUhTP mice deserves a separate study in the future.

Already under sedentary conditions, beige subcutaneous fat tissue is present in the DUhTP mouse model [8], which indicates a larger mitochondrial mass and increased mitochondrial biogenesis in this tissue, further enhanced by training [8,27]. The exercise-induced browning of adipose tissue is associated with increased expression of genes, which are regarded as markers for beige fat as T-box protein 1 (Tbx1), cell death activator (Cidea), or uncoupling protein 1 (Ucp1) [42]. Although physical activity clearly had an effect on fat mass and partly on serum triglycerides in our mouse lines, browning markers were not significantly affected by exercise in the present study. However, an impact of phenotype selection was identified on mRNA expression for Cidea, Tbx1, and Ucp1 by elevated expression in the subcutaneous fat of DUhTP mice. Jash and colleagues described a strong association between CIDEA and *UCP1* in human fat samples [43], which we could also confirm in subcutaneous fat samples from both lines. Surprisingly, *Ppargc1a* (gene for PGC1-α) transcription was unaffected by exercise or selection in the subcutaneous fat depot of DUhTP mice. In previous studies in younger animals, we could detect an increase in the expression of Ppargc1a [8] and Ucp1 [27] in the subcutaneous fat of DUhTP mice. Other studies also demonstrated increased Ppargc1a mRNA expression in subcutaneous fat in response to physical activity [3,5,6,7,9]. However, our previous results were also obtained from animals of an earlier generation under semi-barrier conditions before re-establishing the animals in the current SPF facility [23]. We also cannot exclude the influence of age since the previous experiments were performed with 10-week-old mice and not with 13-week-old ones. Nevertheless, we could clearly demonstrate the prominent role of PGC1-α in the DUhTP line in response to exercise due to the strong correlation between Ppargc1a expression and various other measured mitochondrial-relevant mRNA transcripts.

In muscle samples of healthy, trained human subjects, a strong correlation between Ppargc1a and Nrf1 or Tfam was demonstrated [44]. The authors also could demonstrate a positive correlation between Ppargc1a and mitochondrial creatine kinase, CoxI, CoxXVb, citrate synthase, and the regulators of mitochondrial dynamics.

In our study, a positive correlation between Ppargc1a and Cyc and Cytb, subunits of the respiratory chain complexes, was detectable in DUhTP animals in response to exercise, with a stronger correlation in mice that exercised until the end of the experiment. Nevertheless, no altered gene expression of the investigated complex subunits could be shown in response to any exercise program. By contrast, a selection-mediated increase in Cytb and Cox4i1 gene expression was identified, which was also visible in elevated mitochondrial DNA in DUhTP compared to DUC mice. Three weeks of exercise reduced the mitochondrial DNA (ratio Cytb/36B4) content in DUhTP sed/ex mice. Notably, this reduced mitochondrial DNA was accompanied by decreased gene expression of the transcription factor Nrf1, which allowed direct identification of possible mediators of the negative effects of physical activity on subcutaneous fat energy expenditure. NRF1 induces the transcription of mitochondrial genes encoded in the nucleus and the transcription factor Tfam, which in turn is responsible for the expression of mitochondria-encoded genes [45,46]. NRF1 binds to the promoter of *Cyc* but not to the promoter of the *Cox4i1* gene [47]. Its transcription is induced by NRF2 binding, which we have not investigated in this study. While Nrf1 expression did not correlate with either Ppargc1a, Tfam, or Cyc expression in either line, a positive correlation between Ppargc1a and Tfam was observed in exercised DUhTP animals. Accordingly, neither reduced Nrf1 expression nor reduced mitochondrial DNA levels were correlated with the altered expression of respiratory chain subunits.

In response to exercise, reduced expression of known regulators of mitochondrial fusion and fission was observed in the subcutaneous fat from only DUhTP mice. In comparison to the DUC mice, the reduction found in DUhTP mice can be interpreted as normalization of previously elevated levels, which was significant for Mfn2 in the sedentary condition. Accordingly, a negative effect of exercise on Dnm1 and Mfn2 expression was demonstrated in the DUhTP but not DUC mice. While Dnm1, which mediates GTP-dependent mitochondrial fission [48], was reduced by each exercise intervention in the subcutaneous fat of DUhTP mice, fusion-relevant Mfn2 was reduced in DUhTP mice exercised for three weeks and Mfn1 only in DUhTP sed/ex mice. Interestingly, only the double knockout of muscular *Mfn1* and *2* significantly reduced running performance [49]. Optic atrophy protein 1 (OPA1), which is involved in inner mitochondrial membrane fusion, positively affects exercise performance as provided in Opa1 transgenic mice [50]. Its expression did not vary in response to physical activity in either DUhTP or DUC mice. In comparison, the overexpression of mitofusin-2 in rats did not reveal insights into mitochondrial energy metabolism by using primary skeletal muscle cells [51]. The muscular overexpression of Drp1, instead, reduced muscle growth and attenuated long-term low-intensity running as well as a short-term high-intensity exercise in mice [52]. The overexpression of Fis1 resulted in elevated mitochondrial fragmentation and the simultaneous blocking of fusion [53,54].

In mouse muscle, 30-days wheel running did not change the levels of proteins involved in mitochondrial fusion and fission [23]. Instead, the authors demonstrated that exercise increased phosphorylation at Ser637 of DRP1 (gene Dnm1), inhibiting its translocation to the mitochondria and, thus, reducing mitochondrial fission [23,55]. In male cyclists, reduced mitochondrial fission in response to physical exercise was also concluded based on the increased expression of Ppargc1a, Mfn1 and 2, and Nrf2 in muscle biopsies [56]. However, the muscular expression of genes for mitochondrial fission and fusion may also be related to repair and adaptation after exercise. Accordingly, in rats, 24 h after exercise, a decreased muscular MFN1 protein, increased Fis1 mRNA and protein expression were observed [19,20], and, in mice, a DRP1 activation was induced [21].

This prevailing fission was discussed in the context of degradation of damaged mitochondria before the synthesis of new mitochondria can occur during recovery, which finally leads to an improved mitochondrial quality [18]. Trewin and colleagues recently discussed partially controversial results of mitochondrial dynamics after diverse exercise interventions [22]. Although different energy metabolic responses in the muscle might be attributed to distinct experimental design (intensity, training status, species, sex, age, etc.), there was substantial evidence for an increase of fusion proteins in response to prolonged exercise. The concept of mitochondrial repair and replacement clearly suggests the impact of training intensity and duration on mitochondrial fission and fusion as well as the relevance of the time points when these genes are assessed. The principles of mitochondrial dynamics in muscle might also be relevant for subcutaneous fat, which to our knowledge, has not been studied in this context before.

In fact, in subcutaneous fat, higher mitochondrial fusion in DUhTP mice can be hypothesized due to the reduced expression of the cleavage-relevant Dnm1 in response to long-term physical activity. This assumption would confirm the concept of Trewin et al. [22] now in subcutaneous fat. Although it is impossible to identify the specific functions of the effects of physical exercise on mitochondrial fission and fusion at present, our first mRNA-based results suggest imbalanced mitochondrial dynamics in the subcutaneous fat from our marathon mouse model. Future studies will have to assess the regulation of mitochondrial fission and fusion at the protein level and additional time points to establish a more comprehensive picture of mitochondrial dynamics in subcutaneous fat from DUhTP mice. Another issue for a future study is to test mitochondrial dynamics in subcutaneous fat in response to higher exercise intensities such as forced training.

Nevertheless, the reduced amount of mitochondrial DNA in DUhTP mice due to physical exertion compared to unselected controls can also be interpreted as the normalization of mitochondrial content in subcutaneous fat. Similarly, reduced gene expression associated with mitochondrial fission and fusion could be considered as a normalization with respect to unselected controls.

Furthermore, the substantially reduced subcutaneous fat mass due to physical activity in both mouse lines indicates significant reconstruction activities due to physical activity. However, sedentary DUhTP mice are characterized by elevated levels of energy metabolism in this tissue, which was associated with higher surface temperatures [27]. An explanatory approach for why energy metabolism is increased in subcutaneous fat from marathon mice was not available at that time. The new insights from the present study, with longer durations of the physical activity and later time points of biomarker assessment, may suggest normalization of energy metabolism in subcutaneous fat and support current concepts of interaction between muscle and fat in DUhTP vs. DUC mice [57]. This may fuel the idea that elevated energy metabolism in the subcutaneous fat from sedentary DUhTP mice is related to physical inactivity. This newly established concept will have to be investigated in the future by longitudinal studies addressing the effects of higher training intensities and longitudinal observations. The present study has several limitations. First, the assessment of mitochondrial energy metabolism was performed on mRNA level only and needs to be compared with protein levels and morphology in the future. Also, the timing of adaptive response in subcutaneous fat and the duration of physical activity were predefined by the experimental design. Accordingly, it will be of particular interest for the development of the animal model to study the prolonged effects of physical activity on mitochondrial biogenesis in adipose tissues.

## 5. Conclusions

The present study was performed in order to study the effects and interactions of genotype and voluntary physical activity on the control of energy metabolism in subcutaneous fat from mice. It was possible to confirm previous results showing significant differences in genetic markers for browning and oxidative phosphorylation in DUhTP mice in response to selection. These selection-derived genetic markers were not further induced by the exercise duration and intensity chosen in the current study. Instead, fat mass reductions, accompanied by reduced mitochondrial content, the reduced expression of Nrf1 mRNA, and the reduced expression of mRNA coding for DNM1, MFN1, and MFN2, were observed in response to physical activity. On the level of gene expression, our results demonstrate that the mitochondrial energy metabolism can be triggered by voluntary physical activity in subcutaneous fat from marathon mice but not in unselected controls. This animal model thereby may provide unique opportunities for the study of voluntary activity on adipose tissues.

## Figures and Tables

**Table 1:** List of primers used for mRNA expression analysis in the subcutaneous tissue.

Gene	Forward 5′-3′	Reverse 5′-3′
*Nrf1*	ACAGATAGTCCTGTCTGGGGAAA	TGGTACATGCTCACAGGGATCT
*Tfam*	AAGACCTCGTTCAGCATATAACATT	TTTTCCAAGCCTCATTTACAAGC
*Atp5b*	CGTGAGGGCAATGATTTATACCAT	TCCTGGTCTCTGAAGTATTCAGCAA
*Cyc*	ATAGGGGCATGTCACCTCAAAC	GTGGTTAGCCATGACCTGAAAG
*Cox4i1*	TGGGACTATGACAAGAATGAGTGG	TTAGCATGGACCATTGGATACGG
*18S*	CTGCCCTATCAACTTTCGATGGTAG	CCGTTTCTCAGGCTCCCTCTC
*Cyb*	CTTCGCTTTCCACTTCATCTTACC	TTGGGTTGTTTGATCCTGTTTCG
*36B4*	AGGATATGGGATTCGGTCTCTTC	TCATCCTGCTTAAGTGAACAAACT
*Ucp1*	GGCCTCTACGACTCAGTCCA	TAAGCCGGCTGAGATCTTGT
*Pgargc1a*	CTGCATGAGTGTGTGCTGTG	GGAAGATCTGGGCAAAGAGG
*Tbx1*	GGCAGGCAGACGAATGTTC	TTGTCATCTACGGGCACAAAG
*Cidea*	GTACTCGGTGTCCTACGACATC	TCATCTGTGCAGCATAGGACATA
*Dnm1*	CGGTTAGACAGTGCACCAAG	GGATGTGGGTGGTCACAAT
*Fis1*	GCCCCTGCTACTGGACCAT	CCCTGAAAGCCTCACACTAAGG
*Mfn1*	AGCCAAGGAAGTTCTCAACTC	GCTCTGATAGTGTGCTGTTCA
*Mfn2*	GCCAGCTTCCTTGAAGACAC	GCAGAACTTTGTCCCAGAGC
*Opa1*	TGACAAACTTAAGGAGGCTGTG	CATTGTGCTGAATAACCCTCAA
*Rpl19*	CAATGCCAACTCCCGTCAGC	TCTTGGATTCCCGGTATCTC
*Pgk1*	CAGTCTAGAGCTCCTGGAAGGT	GCCACTAGCTGAATCTTGCG
*Actb*	TGACAGGATGCAGAAGGAGA	CGCTCAGGAGGAGCAATG
*Rplp2*	GACGATGATCGGCTCAACAAG	ACCCTGAGCGATGACATCCT
*Sdha*	CAAATTCTCTCTTGGACCTTGTAGT	CCTTAATTGAAGGAACTTTATCTCCA

*Nrf1*, nuclear respiratory factor 1; *Tfam*, mitochrondrial transcription factor A; *Atp5b*, ATP synthase subunit beta; *Cyc*, cytochrome C; *Cox4i1*, cytochrome c oxidase subunit 4 isoform 1; *Cyb*, cytochrome B; *Ucp1*, uncoupling protein 1; *Ppargc1a*, peroxisome-proliferator-activated receptor-gamma-coactivator-1-alpha; *Tbx1*, T-box protein 1; *Cidea*, cell death activator; *Dnm1*, dynamin related protein 1; *Fis1*, mitochondrial fission 1 protein; *Mfn1*, mitofusin 1; *Mfn2*, mitofusin 2; *Opa1*, optic atrophy protein 1; *Rpl19*, 60S ribosomal protein L19; *Pgk1*, phosphoglycerate kinase 1; *Actb*, actin beta; *Rplp2*, 60S acidic ribosomal protein P2; *Sdha*, succinate dehydrogenase.

**Table 2:** Correlation studies between the expression of transcription factors and genes related to oxidative phosphorylation and mitochondrial fusion (n = 8).

		DUhTP				DUC			
Correlation		sed/sed	sed/ex	ex/sed	ex/ex	sed/sed	sed/ex	ex/sed	ex/ex
Ucp1/Cidea	r	**0.754**	**0.785**	**0.935**	0.48	**0.968**	0.664	**0.865**	**0.793**
	p	*	*	**	ns	***	ns	**	*
Ppargc1a/Nrf1	r	0.375	0.379	−0.266	0.334	0.098	−0.237	−0.444	0.057
	p	ns	ns	ns	ns	ns	ns	ns	ns
Ppargc1a/Tfam	r	−0.000	**0.867**	**0.828**	**0.850**	0.211	0.753	0.008	0.424
	p	ns	**	*	**	ns	*	ns	ns
Ppargc1a/Cyc	r	−0.102	**0.733**	**0.836**	**0.843**	0.275	0.501	0.210	0.021
	p	ns	*	*	**	ns	ns	ns	ns
Ppargc1a/Cytb	r	0.067	0.632	**0.731**	**0.854**	0.406	0.495	0.653	0.211
	p	ns	ns	*	**	ns	ns	ns	ns
Ppargc1a/Mfn1	r	−0.401	**0.866**	**0.925**	**0.945**	0.285	0.626	**0.745**	0.315
	p	ns	**	**	***	ns	ns	*	ns
Ppargc1a/Opa1	r	0.363	**0.943**	**0.788**	**0.899**	0.196	**0.694**	0.414	0.310
	p	ns	***	*	**	ns	*	ns	ns
Mfn1/Opa1	r	0.464	**0.963**	**0.938**	**0.936**	**0.899**	**0.990**	**0.830**	**0.914**
	p	ns	***	**	***	***	***	*	**

Significant correlations were calculated by Pearson correlation, are expressed in bold letters and indicated with two-tailed p-value * p < 0.05; ** p < 0.005; *** p < 0.0005. r, Pearson correlation coefficient; ns, not significant; Ucp1, uncoupling protein 1; Cidea, cell death activator; Ppargc1a, peroxisome-proliferator-activated receptor-gamma-coactivator-1-alpha; Nrf1, nuclear respiratory factor 1; Cyc, cytochrome C; Cytb, Cytochrome B; Mfn1, Mitofusin 1; Opa 1, optic atrophy protein 1; Tfam, mitochondrial transcription factor A.

## Supplementary Materials

The following are available online at https://www.mdpi.com/2073-4409/9/12/2697/s1, Figure S1: Effect of different exercise protocols on the mitochondrial network in subcutaneous adipose tissue of DUhTP (upper row) and DUC mice (lower row).
