# Supplementary material for: Analysis of Activity-Dependent Energy Metabolism in Mice Reveals Regulation of Mitochondrial Fission and Fusion mRNA by Voluntary Physical Exercise in Subcutaneous Fat from Male Marathon Mice (DUhTP)
Source: Cells. 2020 Dec 16;9(12):2697. doi: 10.3390/cells9122697 (PMC7765678; doi:10.3390/cells9122697)
Supplement: Supplementary file 1 [file cells-09-02697-s001.pdf]

cells

MDPI

Article

Analysis of Activity-Dependent Energy Metabolism in Mice Reveals Regulation of Mitochondrial Fission and Fusion mRNA by Voluntary Physical Exercise in Subcutaneous Fat from Male Marathon Mice (DUhTP)

Julia Brenmoehl [1], Daniela Ohde [1], Christina Walz [1], Martina Langhammer [2], Julia Schultz [3] and Andreas Hoeflich [1,*]

1 Institute for Genome Biology, Leibniz-Institute for Farm Animal Biology (FBN); brenmoehl@fbn-dummerstorf.de (J.B.); ohde@fbn-dummerstorf.de (D.O.); walz@fbn-dummerstorf.de (C.W.)
2 Lab Animal Facility, Leibniz-Institute for Farm Animal Biology (FBN), Wilhelm-Stahl-Allee 2, 18196 Dummerstorf, Germany; martina.langhammer@fbn-dummerstorf.de
3 Institute of Medical Biochemistry and Molecular Biology, University of Rostock, Schillingallee 70, Rostock, Germany; julia.schultz@med.uni-rostock.de
* Correspondence: hoeflich@fbn-dummerstorf.de; Tel.: +49(0)38208-68-744

Received: 11 November 2020; Accepted: 12 December 2020; Published: date

Supplementary Materials

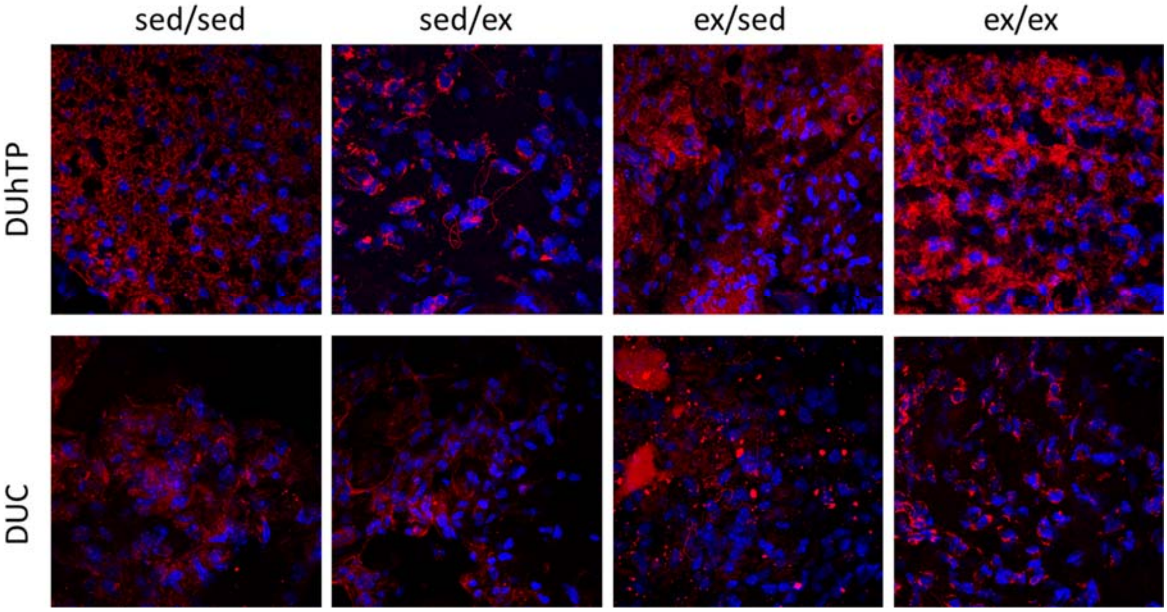

Figure S1. Effect of different exercise protocols on the mitochondrial network in subcutaneous adipose tissue of DUhTP (upper row) and DUC mice (lower row). In a pilot study (n=1), mitochondria were visualized using Mitotracker Deep Red, and cell nuclei were DAPI-stained in cryo-sectioned tissues of both lines, as described in 'Materials and Methods'.
